# Spontaneous transient size reduction of a solitary pulmonary metastasis from a leiomyosarcoma

**DOI:** 10.1186/s40792-023-01591-1

**Published:** 2023-01-26

**Authors:** Nao Ito, Shuhei Iizuka, Kanji Sasaki, Yoshiro Otsuki, Toru Nakamura

**Affiliations:** 1 Department of General Thoracic Surgery, Seirei Hamamatsu General Hospital, 2-12-12 Sumiyoshi, Naka-ku, Hamamatsu, Shizuoka 430-8558 Japan; 2 Department of Orthopedic Surgery, Seirei Hamamatsu General Hospital, 2-12-12 Sumiyoshi, Naka-ku, Hamamatsu, Shizuoka 430-8558 Japan; 3 Department of Pathology, Seirei Hamamatsu General Hospital, 2-12-12 Sumiyoshi, Naka-ku, Hamamatsu, Shizuoka 430-8558 Japan

**Keywords:** Solitary pulmonary nodule, Leiomyosarcoma, Pulmonary metastasis

## Abstract

**Background:**

A solitary pulmonary nodule (SPN) poses a diagnostic challenge, which includes both a benign and malignant etiology. A size enlargement often indicates malignancy. We herein describe a case of a solitary pulmonary metastasis from a leiomyosarcoma that regressed transiently during follow-up.

**Case presentation:**

A 47-year-old woman presented with an SPN detected by follow-up computed tomography 7 years after surgery for a left forearm high-grade leiomyosarcoma. The nodule regressed spontaneously after an additional 6 months, and therefore, an inflammatory change was the most likely diagnosis at that time. However, the nodule enlarged again over the next 5 years. The growth rate led us to suspect a malignancy. A trans-bronchial biopsy was undiagnostic and a video-assisted thoracic surgery was planned. She underwent a wedge resection of the right lung, and a histopathological examination found it was a metastatic leiomyosarcoma.

**Conclusions:**

A pulmonary metastasis from a leiomyosarcoma could emerge as an SPN and reveal a subsequent transient size reduction. An SPN in patients even with a remote history of a soft tissue tumor should raise the possibility of metastasis, and periodic follow-up is essential even after the size reduction.

## Background

A solitary pulmonary nodule (SPN) poses a diagnostic challenge, which includes both a benign and malignant etiology. A size enlargement often indicates a malignancy that could contain both primary and metastatic etiologies. While most metastatic pulmonary lesions often manifest as multiple onsets, a solitary metastasis could also develop. We herein describe a case of a solitary pulmonary metastasis from a leiomyosarcoma that regressed transiently during a prolonged follow-up of over a decade.

## Case presentation

**Fig. 1:**
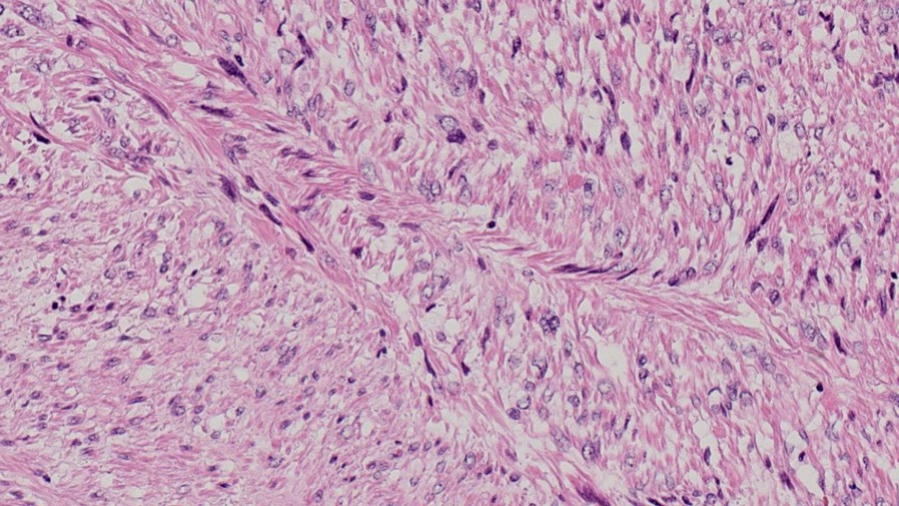
A histopathological examination of the left forearm high-grade leiomyosarcoma showing dense proliferation of spindle-shaped cells with an eosinophilic cytoplasm and cellular atypia

**Fig. 2:**
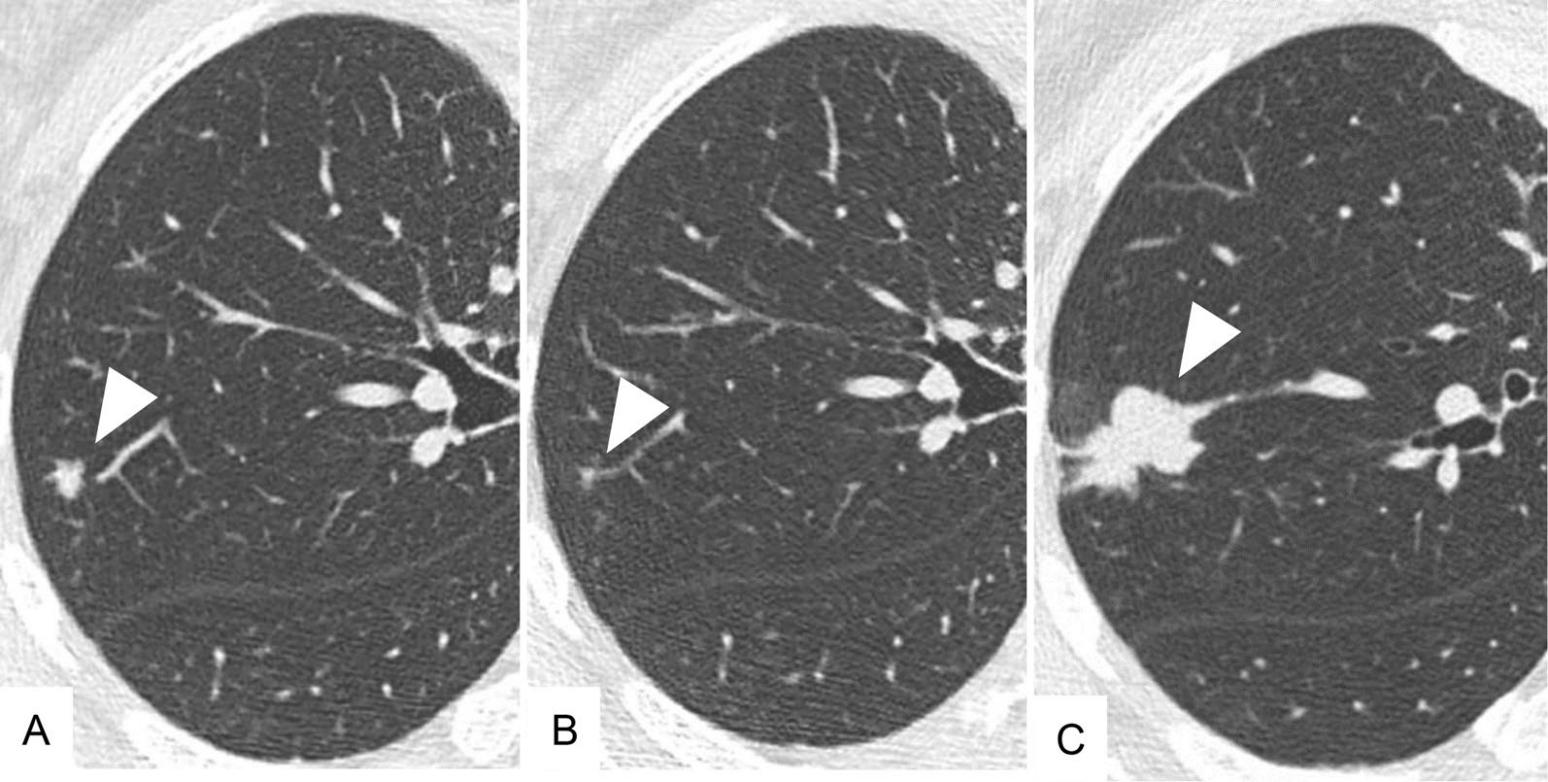
**A** A chest CT 7 years after radical surgery for the leiomyosarcoma showed a 4-mm irregular nodule (arrow). **B** The nodule regressed spontaneously to 2 mm after an additional 6 months. **C** The nodule regrew up to 24 mm over the next 5 years

**Fig. 3:**
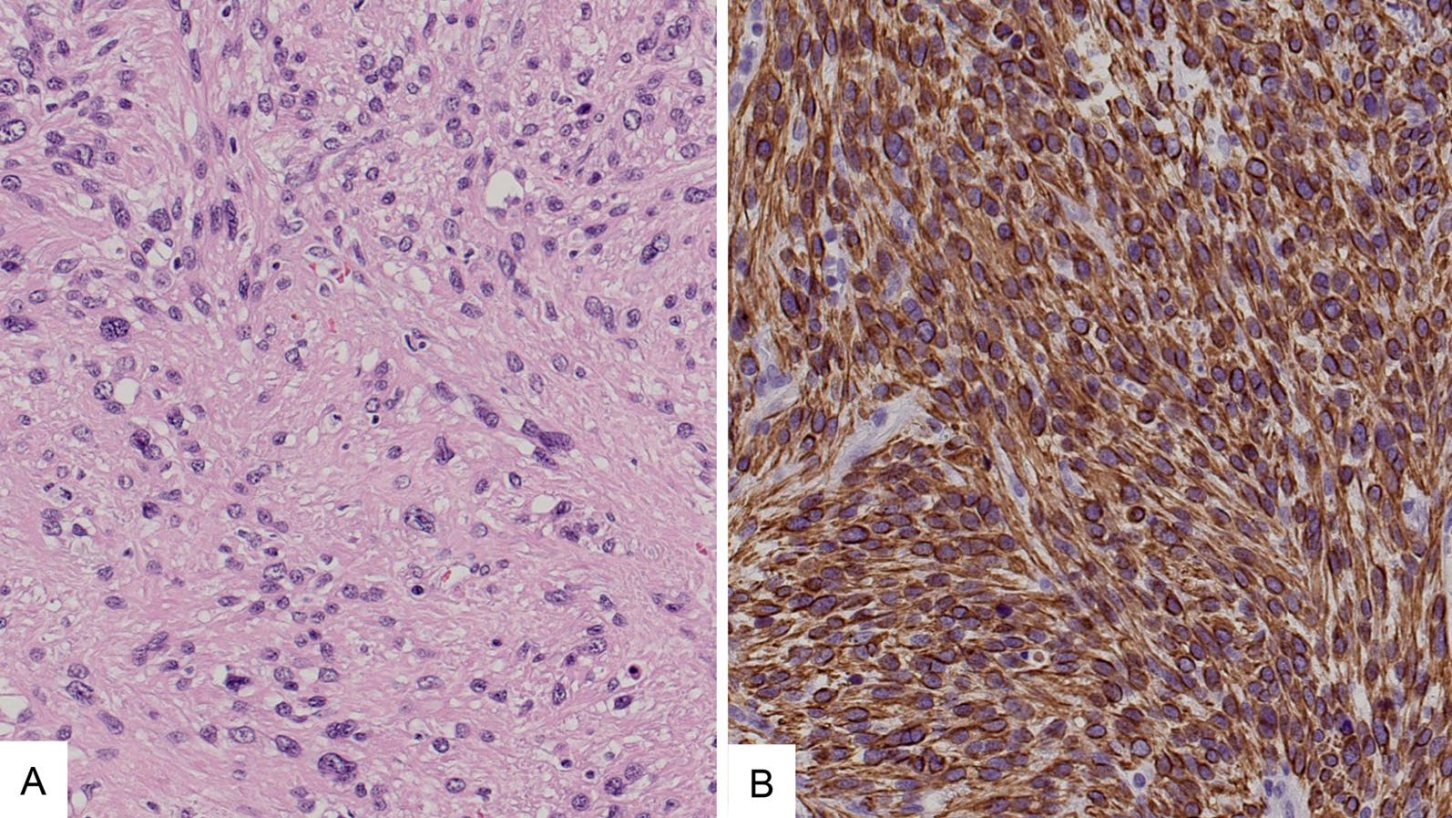
**A** A histopathological examination of the lung nodule showing dense proliferation of spindle-shaped cells with an eosinophilic cytoplasm. **B** The tumor cells were immunohistochemically positive for desmin and were consistent with a metastatic leiomyosarcoma

A 47-year-old woman presented with an SPN detected by follow-up computed tomography (CT) 7 years after surgery for a left forearm high-grade leiomyosarcoma (Fig. 1). A chest CT showed a 4-mm irregular nodule in the right upper lobe (Fig. 2A), which enlarged to 6 mm 2 months later. The nodule spontaneously regressed to 2 mm 4 months later, and therefore an inflammatory change was the most likely diagnosis at that time (Fig. 2B). However, the nodule enlarged again up to 24 mm over the next 5 years (Fig. 2C) and led us to suspect a malignancy. Primary lung cancer was more likely than a metastasis because of the solitary spiculated appearance and time interval of 12 years from the leiomyosarcoma surgery [1–3]. A trans-bronchial biopsy was undiagnostic and a video-assisted thoracic surgery was planned. She underwent a wedge resection of the right lung, and a histopathological examination revealed a dense proliferation of spindle-shaped cells with a marked pleomorphism and mitoses (Fig. 3A). The tumor cells were stained positively by desmin and were consistent with a metastatic leiomyosarcoma (Fig. 3B). The permanent histologic margin was negative. We did not investigate the biomarker status including programmed death ligand 1. Postoperative positron emission tomography CT showed no other organ involvement. She is currently disease free at 15 months after the lung surgery.

## Discussion

The lungs are the most common distant metastatic site from a leiomyosarcoma [4]. A pulmonary resection is a feasible treatment option in selected patients and favorable prognostic factors include a longer disease-free interval (DFI), smaller number of metastases, and a complete resection [5, 6]. The present case is expected to have a good prognosis because of the complete resection of the solitary metastasis with a 7-year DFI. Longer and more frequent follow-ups also contributed to the favorable outcome.

The present case suggested two important issues. First, a pulmonary metastasis from a leiomyosarcoma could manifest as an SPN and subsequently reveal a transient size reduction. An SPN poses a diagnostic challenge because of the wide range of the pathogenesis, including benign and malignancies. Besides the larger size, the growth rate is also suggestive of a malignancy, and therefore periodical radiological follow-ups are essential [7, 8]. Although the pulmonary nodule in the present case enlarged during the first 2 months, it spontaneously regressed thereafter. We suspected the nodule was an inflammatory change, but it consequently grew and was found to be a metastasis 12 years after the initial surgery. The cause of the transient size reduction was not clear; however, several mechanisms such as inflammation, infections, and autoimmune activation have been reported to be responsible in the literature [9–11].

Second, a pulmonary metastasis from a leiomyosarcoma could emerge in the remote period after the initial treatment. The current guidelines for the management of a soft tissue sarcoma recommend periodical follow-ups every 3 or 4 months for 2 or 3 years, twice a year until the 5th year, and once a year thereafter [12]. A pulmonary metastasis emerged 7 years after the initial surgery in the present case and might have been overlooked without a strict guideline-compliant follow-up. The prolonged observation even after the size reduction also contributed to the successful outcome. These results also highlight the validity of the current guidelines.

## Conclusions

A pulmonary metastasis from a leiomyosarcoma could emerge as an SPN and transiently regress with an unknown mechanism. An SPN in patients even with a remote history of a soft tissue tumor should raise a suspicion of a metastasis and periodic follow-ups are essential even after a size reduction.

## Data Availability

Not applicable.

## Abbreviations

CT: Computed tomography
SPN: Solitary pulmonary nodule
